# Intuition-Talk: Virus or Virtue?

**DOI:** 10.1007/s11406-016-9796-6

**Published:** 2017-01-22

**Authors:** James Andow

**Affiliations:** Department of Philosophy, University of Reading, Reading, UK

**Keywords:** Philosophical methods, Intuition, Hedging, Metaphilosophy, Epistemic humility, Intellectual humility

## Abstract

The word ‘intuition’ is used frequently both in philosophy and in discussions about philosophical methods. It has been argued that this intuition-talk makes no (clear) semantic contribution and that intuition-talk is thus a bad habit that ought to be abandoned. I urge caution in making this inference. There are many pragmatic roles intuition-talk might play. Moreover, according to one plausible story (for which there is some empirical support), there is reason to think intuition-talk is actually a good habit for philosophers to have.

## Introduction

The word ‘intuition’ is used frequently both in philosophy and in discussions about philosophical methods. A growing number of philosophers such as Cappelen (2012) and Williamson (2007) hold that this intuition-talk is a bad habit that ought to be abandoned.^1^

As Cappelen pithily remarks,

Philosophers’ use of ‘intuition’ is a kind of intellectual/verbal virus (or tick) that started spreading about thirty to forty years ago. It is a bad habit and we should abandon it. Cappelen (2012, p. 50)

Why think intuition-talk is a bad habit? One main reason goes something like this: intuition-talk is semantically defective (in the sense that ‘there’s no semantic anchor point and the term [‘intuition’] fails to have a semantic value’, Cappelen 2012, p. 50); so, we should stop using intuition-talk.^2^ For the purposes of this paper, I won’t be questioning the starting point. Suppose it is true that intuition-talk is semantically defective. My main point is to note that this doesn’t mean that intuition-talk is a bad habit. Even if intuition-talk makes no *semantic* contribution, it doesn’t follow that it is a bad habit. If intuition-talk makes a helpful *pragmatic* contribution, then it seems appropriate to deem it a good habit.

So what pragmatic roles might intuition-talk play? There are many. I won’t explore them all in detail here. But let me mention a few to illustrate. (1) Intuition-talk might play the role of marking aspects of the dialectic, e.g., ‘intuitively, p’ might pragmatically communicate, ‘you may or may not accept p, but please the conversation I am interested in having holds p fixed’, or ‘p is to be challenged before some other claim q’, or ‘if you don’t accept p, then the conversation is about to get rather more complicated’, or ‘p is the weakest part of my argument’. (2) Intuition-talk might invite interlocutors to consider a claim. (3) Intuition-talk might raise a question, ‘is there any support for p?’(4) Intuition-talk might straightforwardly strengthen the force of an assertion. (5) Intuition-talk might straightforwardly weaken the force of an assertion.

Before we can write off semantically-defective intuition-talk as a bad habit, we need to first ask whether intuition-talk in fact plays any of these pragmatic roles in philosophy and, if so, whether that pragmatic contribution is a bad one or a helpful one. This is a project which the current paper can’t hope to complete in its entirety. Rather, I will explore one specific variety of the final proposal from the previous paragraph. I will make the case that intuition-talk in the academy is plausibly associated with a known rise in ‘shielding’ language in the academy, and that intuition-talk (if it plays a hedging role) plausibly makes a positive contribution to academic philosophy.

## Intuition-talk is on the Rise (and Philosophers aren’t Special)

What do we know about intuition-talk in philosophy and the academy more widely?

First, *philosophers* use the word ‘intuition’ a lot more than they used to (see Andow 2015a). This fact is widely recognized in the debate about the role of intuitions in philosophy (see Goldman 2007; Hintikka 1999). There is a surprise lurking, however: philosophers are not alone. In recent quantitative empirical work I have shown that intuition-talk exploded across a great swathe of academia, and use of words like ‘intuition’ even seems to have increased in non-academic writing, such as fiction and journalism (Andow 2015a).^3^

Second, this finding provides some reason to think that philosopher’s use of intuition-talk isn’t special. Since this explosion of intuition-talk is a general phenomenon, the best explanation for it, then, must be largely general as well.^4^

Third, this picture, according to which there is no special use of intuition-talk among philosophers, is supported by my recent qualitative work that compares intuition-talk in philosophy with intuition-talk elsewhere (Andow 2015b).

Cappelen notes that in ordinary English when ‘intuition’ and ‘intuitive’ are used to modify propositions (which is not so frequently), their function is to serve as a *hedge*. There is reason to think a hedging function of ‘intuitive’ may be bound up with the rise of intuition-talk in the academy—so let’s take a closer look.

## Hedging

What is a hedge? Lakoff (1975), who coined the term ‘hedge’, puts it as follows: hedges ‘make things fuzzier’. Hedging expressions include, ‘it seems that’, ‘one might conclude that’, ‘I think’, ‘kind of’, ‘approximately’, ‘it could be suggested that’ and so on. As these examples indicate, hedges are a somewhat heterogeneous class. One important distinction is that between *shields* and *approximators*:^5^

- *Shields*: hedges relating to the standing of the proposition (e.g., concerning probability, or evidential standing of the proposition)
- *Approximators*: hedges relating to the content of the proposition. A typical example of an approximator is ‘sort of’, e.g., in ‘well-being *sort of* benefits from a temperate climate’.

Note that hedging intuition-talk typically seems to shield rather than approximate. For example, in ‘intuitively, my well-being benefits from a temperate climate,’ the word ‘intuitively’ is used to ‘back off’ the straight assertion, it indicates a weakened commitment to the proposition.^6^

What do we know about hedges in the academy? To date, studies of hedging in academic texts reveal some notable patterns which suggest a possible relation between intuition-talk in philosophy and shielding.^7^

1. Cross-disciplinary studies find that philosophy is among the fields that make greatest use of hedging (Hyland 1998, 2001; Hyland and Salager-Meyer 2008, and Hyland and Tse 2004). (See also Gómez et al. (1998) and Poos and Simpson (2002) for some other interdisciplinary observations).
2. Diachronic studies find that use of *shielding* in particular has increased (in much the same way that Andow (2015a) finds that intuition-talk has increased in the academy).
  - The clearest evidence comes from Salager-Meyer and Defives (1998) who note that across publications in medical periodicals (including, e.g., reviews and editorials), ‘[s]ince 1930, and especially since 1950, the ‘shield’ … became more frequently used’ (p. 133). It is not possible to provide a clear figure based on Salager-Meyer and Defives’s graphs (p. 148–149), but, roughly, the rate of hedging per 100 words seems to have doubled (from around 3 to around 6) in the period 1930–1995. Focusing on research papers, the authors note ‘the closer we get to the turn of the twenty-first century, the more [research papers] seem to slide towards the SH [i.e., *shielding*] variable’ (p. 154).
  - Although, to my knowledge, no researchers have yet examined shielding across the full spectrum of academia (see e.g., Alonso-Almeida and Marrero-Morales 2011 comments in their introduction to a special issue on Diachronic English for Specific Purposes), Gross et al. (2002, p. 231) discuss a wider range of disciplines and note similar themes (although their analysis is predominantly qualitative and they don’t explicitly distinguish shielding from other types of hedge).
  - It is important to note that *hedging in general* has not increased. Salager-Meyer and Defives (1998) show that overall academic language becoming slightly less hedged over the C20th. Similarly, Gross et al. (2002, p. 165) show a flat trend throughout the C20th (despite a large increase on the C19th, p. 163).

These patterns suggest that the ‘verbal virus’ which Cappelen observes in philosophy may plausibly be connected with a rise in shielding in the academy. The ascendancy of shielding seems a plausible candidate for being an important factor behind the explosion in intuition-talk.^8^ In other words, there is at least some plausibility to the idea that intuition-talk in philosophy may be associated with the pragmatic function of shielding.

## Virus or Virtue?

Is intuition-talk which plays the pragmatic role of shielding assertions to be lauded or loathed? Is intuition-talk a bad habit or vice? Or is it a good habit or virtue? In this section, I make the case that such intuition-talk is a *good* habit.

Recall that the disparagement of intuition-talk *as a bad habit*, largely rests on the idea that intuition-talk is *semantically defective*, i.e., makes no semantic contribution.^9^ However, even if intuition-talk makes no *semantic* contribution, it doesn’t follow that it is a bad habit.

Indeed, a quick survey of the literature on English for Academic Purposes, suggests that shields, and thereby shielding intuition-talk, serve an *intellectually positive* pragmatic function in academic debate. I will emphasize two themes from this literature, these themes concern aspects of hedging (which are associated in particular with the shielding type of hedging) which seem particularly beneficial to philosophical discourse. The two themes are *politeness* and *intellectual humility*, both of which I consider to be virtues.

First, consider *politeness*. Bald (unshielded) assertions project authority; they claim to capture exactly how things are, setting aside no space for the audience to deliberate or disagree. Shielded assertions are less intrusive and therefore more polite, in the negative sense of not impinging on others’ autonomy (Brown and Levinson 1987; Mihatsch 2012; Myers 1989). As Myers explains,

Hedging is a politeness strategy when it marks a claim, or any other statement, as being provisional, pending acceptance in the literature, acceptance by the community – in other words, acceptance by the readers Myers (1989, p. 12).

Philosophical discussions *benefit* from this kind of politeness. Aggressive, adversarial styles can hinder progress by placing value on the production of clever refutations rather than the pursuit of understanding (Moulton 2003; Stevens 2015).^10^ Using politely shielded speech encourages a more constructive environment, for shields signal a willingness to be questioned and respond to questions in a friendly way. Shielded assertions also make it easier for everyone to engage in the intellectual discussion; it is less emotionally difficult and personally risky to criticize an ‘intuition’ than a straight assertion.

Second, consider *intellectual humility*. By weakening a speaker’s presumption to authority, shielded assertions also signal humility (Hyland 1998; Salager-Meyer 1994; Swales 1990). Many authors draw attention to Robert Boyle (the seventeenth century scientist) who ‘saw hedging as one of many stylistic devices to project both personal honesty and modesty’ (Lewin 1998, citing Shapin 1984 and Swales 1990). Roberts and Wood describe the virtue of intellectual humility as “a very low concern for intellectual domination in the form of leaving the stamp of one’s mind on disciples, one’s field, and future intellectual generations” Roberts and Wood (2003, p. 271). It seems clear that this virtue is good for philosophy. Humble scholars are less likely to be misled by their own arrogance into overestimating their own ideas or underestimating those of others. They are also less likely to avoid exploring potentially unpopular ideas for fear of wounding their vanity Roberts and Wood (2003, p. 271).

My main point in this paper is to highlight that even if philosophers’ intuition-talk is a semantically empty symptom of a verbal virus, this doesn’t mean that it is a vice. It may play a pragmatic role and that pragmatic role may be a positive thing. To that end, the example of the virtues of shielding is simply a hypothetical example. However, I do think that this is a promising avenue to explore further since, for the reasons given above, it is plausible that intuition-talk in the academy and the use of shielding language may be associated. So there is hope yet that, far from being a vice, intuition-talk may be a welcome feature of philosophical discourse.^11^ Shields are polite and a sign of intellectual humility; as a way of shielding, intuition-talk is too.

## Objections and Replies

Quickly, before we finish, let me acknowledge and respond to some likely lines of resistance:

1. *If intuition-talk were just a pragmatic way to shield a statement, it would be interchangeable with phrases like “I think.” Yet “Intuitively, it’s going to rain” doesn’t mean the same thing as “I think it’s going to rain.”*

I didn’t claim that intuition-talk is solely about shielding. In fact, I didn’t even concede Cappelen’s claim that intuition-talk is completely semantically empty.^12^ I am open to the idea that, as this objection suggests, intuition-talk is not purely pragmatic but rather has some kind of semantic anchor. However, this doesn’t affect the argument that intuition-talk has a positive pragmatic role to play.^13^

(2) *If you want to understand philosophers’ intuition-talk, X is a very important factor (and much more important than shielding).*

I can’t deal with all possible iterations of this line of resistance. But I can anticipate and respond to some of the most likely versions, e.g.:

- *In philosophy, we use a technical sense of ‘intuition’ such that describing a claim as ‘intuitive’ actually strengthens it. Intuitive claims aren’t just lacking a certain kind of support; intuitions offer their own kind of respectable, reliable evidence. So, the dominant role of intuition-talk cannot be a form of hedging.*
- *In philosophy, intuition-talk is motivated by an unrealistic desire for non-controversial evidence*. *The idea that intuition-talk often serves to hedge commitment only bolsters the connection between intuition-talk and this illicit desire. Intuition-talk is just a way for the overly timid to preemptively armor their claims by rendering them so weak that they become utterly uncontroversial, acceptable even by skeptics. So, intuition-talk is a virus rather than a virtue.*

Note that these, and many other, versions of the response rely on the idea that philosophers are meaning or doing something special when they engage in intuition-talk—philosophers don’t do as others do. However, as I emphasized above, this is not supported by the evidence. The available empirical evidence (Andow 2015a, b) suggests that philosophical intuition-talk isn’t so special. I agree that philosophers *might* have a unique motivation to use intuition-talk. However, I agree with Andow (2015a) that our understanding of intuition-talk in philosophy needs to be one in which philosophy-specific factors play only a minor role. The major factors must be more general ones. Furthermore, I am inclined to agree with Cappelen (2012) that there is little evidence of any coherent philosophy-specific trends in philosophers’ intuition-talk; there is no settled technical philosophical definition of ‘intuition’ and many philosophers use the word without harboring any substantial commitments as to its meaning. It is thus not plausible that philosophers’ intuition-talk is insulated from general linguistic trends. If we want to understand intuition-talk and its effect on philosophy, it would be amiss to focus solely on philosophy-specific forces.

(3) *You underestimate the confusion caused by the fact that there are so many different understandings of intuition and uses of ‘intuition’ in philosophy. This is an important negative aspect of intuition-talk which renders it a bad habit.*

Perhaps. However, at this point the burden is on the person raising the objection. I am inclined to think that there is no significant confusion caused at the level of first-order philosophy. I agree with Cappelen on this point – directly after the quote I opened with, Cappelen continues, ‘this is important, the virus didn’t have much effect on first-order philosophy’. I am inclined to agree with Cappelen that there is a lot of confusion at the level of methodology, i.e., metaphilosophical theorizing about intuitions (see Andow 2016). However, this is no reason to think intuition-talk is a bad habit which should be abandoned in first-order philosophy.^14^ Methodologists’ ability to clear up their mess is not dependent on whether *intuition*-*talk* gets abandoned at level of first-order philosophy; it isn’t likely that abandoning intuition-talk at the first-order level would be what made methodologists forget their decades’-old debates.

## Footnotes

1 ‘Intuition-talk’ = using terms like ‘intuit,’ ‘intuition(s),’ ‘intuitive,’ ‘counter-intuitive,’ ‘intuitively,’ and ‘counter-intuitively’.

2 Cappelen also thinks it troubling that intuition-talk has misled metaphilosophers into exaggerating the role intuitions play in philosophical methodology. However, even if true, this would not be a good reason to abandon intuition-talk. Methodologists may have made a mistake, but getting first-order philosophers to stop using intuition-talk is neither a necessary nor an effective step towards reorienting methodological discussion. It is not necessary as methodologists could be persuaded rationally with arguments. It wouldn’t be particularly effective as the debates have already started—the prevalence of intuition-talk might have been a catalyst to get them started, but the debates now pay little attention to how philosophers use language. It *might* be more significant were the metaphilosophers Cappelen targets to support their claims about intuition by pointing to the verbal practices of philosophers. But this is not how any of them argue (Malmgren 2013).

3 The rate of the explosion in intuition-talk has been, if anything, slightly lower in philosophy than other disciplines.

4 Of course, it is *possible* for such a general pattern to be produced by a coincidental confluence of individual causes. However, in this case, the scale of the coincidence would be somewhat dramatic, so the most likely explanation is one in which the major factors are general ones. This isn’t to deny that some minor more local factors might have made some minor contribution.

5 This distinction was first made by Prince et al. (1982). Almost all taxonomies of hedges make the same distinction, although nomenclature varies. One might further distinguish between straight shields and those which express an ‘author’s personal doubt and direct involvement’ by using, e.g., ‘I’ (as Salager-Meyer 1994). But, for my purposes, I’ll consider these cases of shielding (as, e.g., Salager-Meyer and Defives 1998).

6 Some eccentric uses of intuition-talk might be approximators, e.g., ‘there’s a very intuitive sense in which p’. However, such uses are unusual. For example, a quick search reveals no use of intuition-talk as an approximator in Philosophical Review Oct 2008 to October 2009. There are some uses of ‘intuitive x’ (attaching intuition-talk directly to a noun phrase, e.g. ‘intuitive difference’ and ‘intuitive cost’ rather than a clause, e.g., ‘intuitively, p’). However, these are not plausibly interpreted as making the content fuzzier.

7 There is some reason to suspect these patterns may be peculiar to academic English (see Salager-Meyer et al. 2007; Salager-Meyer et al. 2003, and Hyland and Salager-Meyer 2008).

8 It seems likely any such tendencies reflect wider social changes. So an explanation of increased shielding in the academy could well also explain the patterns Andow (2015a) observed in Time magazine and the corpus used by Google Ngrams.

9 As noted in footnote 2, Cappelen’s main gripe is that intuition-talk has misled metaphilosophers into exaggerating the role intuitions play in philosophy, but this isn’t a good reason to brand intuition-talk a bad habit.

10 Moulton recommends wholesale changes to current practice, but one needn’t accept her more ambitious proposal to appreciate the point that too much aggression can impede philosophical progress.

11 I should note that not everyone thinks that every aspect of hedging is a positive one. While many see hedging as part of intellectual honesty, some have discussed a worry that hedging might be associated with an unscientific imprecision (Benham et al. 2012, Salager-Meyer et al. 2003, Skelton 1988). However, this doesn’t undermine my case that there is good reason to think intuition-talk is overall a good habit.

12 My strategy has been to show that even *if* intuition-talk is semantically defective, that would still not be any reason to stop using it. I have not pretended to offer any arguments for the antecedent.

13 Indeed, most plausible ideas about any such semantic anchor fit with the idea that intuition-talk serves to shield assertions. Nearly every attempt to define intuitions begins with pointing out that they lack inferential and empirical support, e.g., Chalmers (2014, p. 537). It seems plausible, then, that the term ‘intuitive’ helps calls attention to a kind of support a claim doesn’t have, which is one way to hedge it.

14 See also footnote 2.
